# Nanozymes Regulate Redox Homeostasis in ROS-Related Inflammation

**DOI:** 10.3389/fchem.2021.740607

**Published:** 2021-10-20

**Authors:** Qing Li, Ying Liu, Xianglin Dai, Wei Jiang, Huan Zhao

**Affiliations:** ^1^ Department of Oncology, The First Affiliated Hospital of Zhengzhou, Zhengzhou, China; ^2^ Application Center for Precision Medicine, Department of Molecular Pathology, The Second Affiliated Hospital of Zhengzhou University, Zhengzhou, China; ^3^ Center for Precision Medicine, Academy of Medical Sciences, Zhengzhou University, Zhengzhou, China

**Keywords:** nanozyme, inflammatory, brain diseases, redox homoeostasis, ROS

## Abstract

Reactive oxygen species (ROS), in moderate amounts, play an essential role in regulating different physiological functions in organisms. However, increased amounts of ROS may cause oxidative stress and damage to biomolecules, leading to a variety of diseases including inflammation and even cancer. Therefore, ROS scavenging reagents are needed to maintain healthy levels of ROS. With considerable advances in nanotechnology, nanozymes possess SOD or CAT-like activities with outstanding free radical scavenging activity, facile synthesis conditions, and excellent biocompatibility. Based on these extraordinary properties, nanozymes has been used to modulate the redox homeostasis and relieve the ROS-related injury. This has led to the emergence of nanozyme-based therapies. In the current review, we presented recently developed applications of nanozymes to treat ROS-dependent disorders with an emphasis on inflammatory and brain diseases.

## Introduction

Reactive oxygen species (ROS) are molecules formed due to incomplete reduction of O_2_, which is a well-known process in the fields of chemistry or biology. In a nutshell, ROS are highly reactive substances that contain oxygen. They include superoxide anions (˙O2^−^), hydrogen peroxide (H_2_O_2_), hydroxyl radicals (˙OH), singlet oxygen (^1^O_2_), peroxy radicals (LOO˙), hydrogen peroxide lipids (LOOH), peroxynitrite (ONOO^−^), hypochlorous acid (HOCl), ozone (O_3_), etc (Wang et al., 2011; Li et al., 2016; Muszynska et al., 2020). Over the past century, researchers have tried to unveil the origin of ROS. For example, it was found that OH˙ is generated during the photolysis of ozone. Identifying the processes of the formation of ROS may help utilize ROS by avoiding its ill effects (Bauer et al., 2011).

**FIGURE 1:**
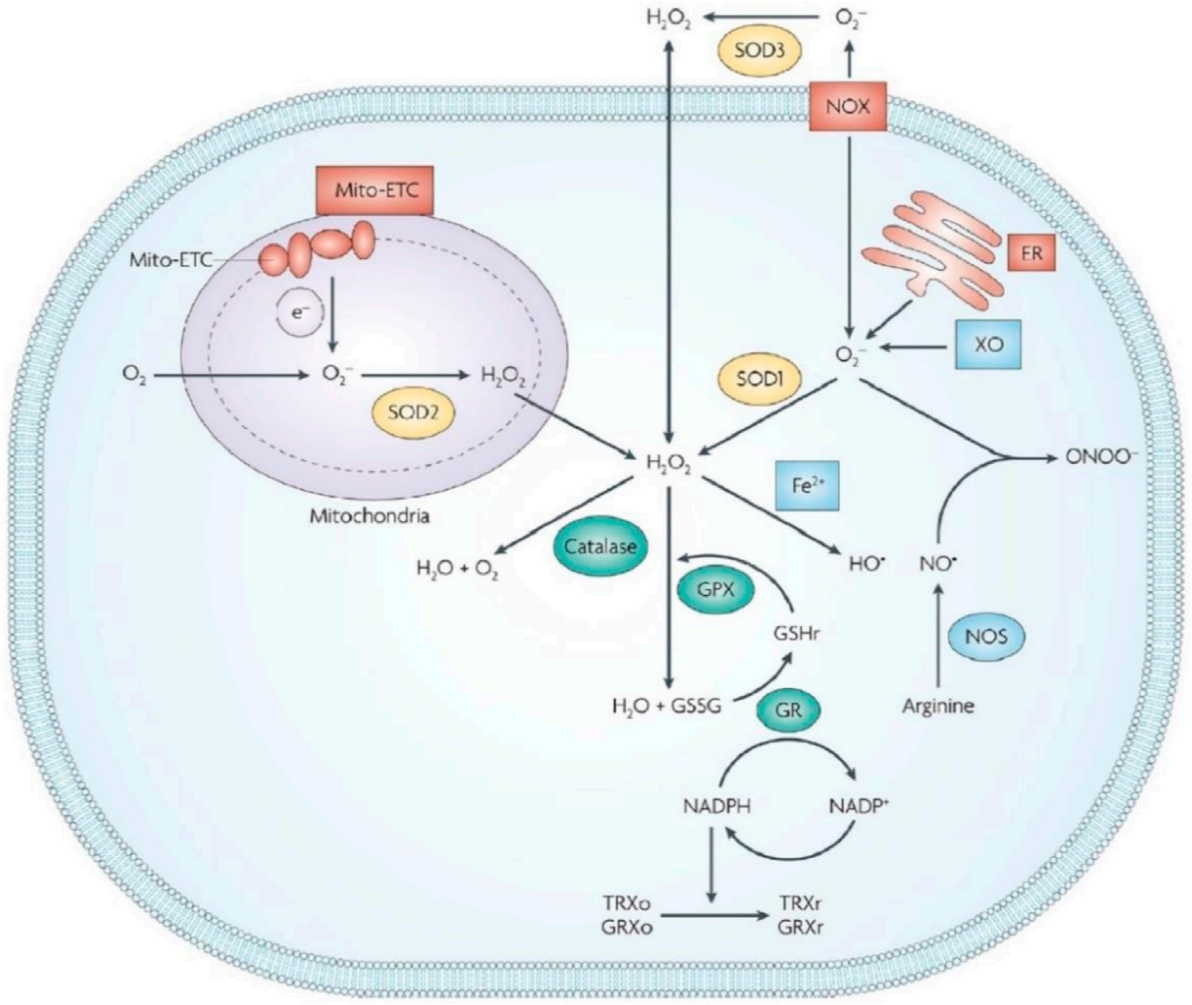
ROS-induced cellular redox homeostasis. Endogenous and exogenous pathway have close relationship with intracellular ROS levels. In order to maintain ROS at a normal level for organism well-being, ROS-ablating enzymes are available in cells to avoid oxidative stress. GR, glutathione reductase; GPX, glutathione peroxidase; GRXo, oxidized glutaredoxin; GRXr, reduced glutaredoxin; TRXo, oxidized thioredoxin; TRXr, reduced thioredoxin; GSHr, reduced glutathione; GSSG, oxidized glutathione. Reproduced from Wu et al. (2019a) with permission from the American Chemical Society.

Organisms are the cardinal producers of ROS using the endogenous and exogenous pathways (Huo et al., 2017). The production of ROS in the mitochondrial respiratory chain using special enzymes such as cyclooxygenases and xanthine oxidase is termed as the endogenous pathway (Trachootham et al., 2009). The increase in the production of ROS in organisms due to radiation, environmental pollution, and other chemicals is defined as the exogenous pathway (Sun et al., 2019). The amount of ROS in an organism depends not only on the amount of ROS produced by the organism but also on the organism’s ability to remove ROS, known as the antioxidant capacity, which involves a series of antioxidant enzymes like superoxide dismutase (SOD), catalase (CAT), etc (Figure 1) (Wu et al., 2019a). ROS is a double-edged sword, which can exert positive effects for several physical activity like wound healing and physiological regulation. Nevertheless, superfluous ROS will cause destructive results. In healthy organisms, ROS maintains a state of equilibrium and uses it to execute its functions. For example, Foreman et al. elaborated on the vital role of nitrogen oxides (NOx) in plant growth *via* ROS (Foreman et al., 2003). Niethammer demonstrated that a gradient of H_2_O_2_ developed around the wound in Zebrafish larvae facilitating wound healing (Niethammer et al., 2009). However, due to stimulation by the environmental agents and diseases, the generation and clearance of ROS become unbalanced, causing oxidative damage if the unbalance is beyond the tolerance thresholds of the organism, which is paralleled with damage to proteins, lipids, and nucleic acids (Nakazawa et al., 2016). Mild oxidative damage leads to changes in cell function and behavior, such as accelerated aging, abnormal proliferation, inflammatory response, etc. and severe oxidative damage may lead to apoptosis and autophagy (Vanzella et al., 2017). Therefore, it is important to maintain redox homeostasis to avoid ROS-dependent diseases, such as cancer, inflammation, radiation damage, and neurological diseases.

Since Fujishima and Honda found that TiO_2_ generates ˙OH by photocatalytic water-splitting in 1972 (Fujishima and Honda, 1972), the exploration for other ROS-producing catalysts for industrial advancement and biomedical treatments has never ceased. Excess production of ROS exacerbates the diseases like cancer and bacterial infections (Karimi et al., 2015). For example, in cancer therapeutics, nanomedicines with specific properties produce ROS photocatalytically and sonocatalytically under the stimulus of light and ultrasound (US), respectively. They are called photodynamic therapy (PDT) and sonodynamic therapy (SDT), respectively. Additionally, some nanoparticles with peroxidase-like activities, such as CuS particles (Xi et al., 2019), MoO_3_ nanodots (Zhang et al., 2021a), and AgPd_0.38_ nanocages (Gao et al., 2021), augment the generation of ˙OH, a cytotoxic ROS, by consuming hydrogen peroxide. They are used in organisms as antibiotics. As a result of the heterogeneity in tumor tissue and multidrug-resistant bacteria, the current clinical research emphasis has shifted from monotherapy to synergistic therapy, in which PDT, SDT, and enzymatic ROS-generation are involved (Karimi et al., 2015). Nanotherapy stimulate the wave of technological innoventions in ROS field, which has benefited the emergence of a variety of nanomaterials. The size effect and surface area are the main advantages of nanomedicines compared with medicines with micro or other dimensions. Specifically, crystallographic transformations will occur on the surface of nanomaterials when the sizes of materials are decreased below 30 nm (Xi et al., 2019). This change will regulate the interfacial reaction kinetics on the surface of nanomaterials. Besides, the large surface areas of nanoparticles will afford plenty of anchoring points for reactive molecules such as ROS, thus enhancing their chemical reactivity. Moreover, the small sizes of nanomedicines can also benefit the cell/tissue uptake and intracellular transport of these nanosystems (Wu et al., 2019a).

Strategies for redox homeostasis, in turn, regulate the redox balance *via* ROS generation and depletion, improving ROS-associated pathological conditions (Petro et al., 2016). With rapid development in nanomaterial science and enzymology, inorganic nanomaterials with enzyme-mimicking properties have been developed to eliminate aberrant ROS for healthy physiological functioning (Wu et al., 2019b). In 2007, Chinese scientists discovered that Fe_3_O_4_ nanoparticles had horseradish peroxidase (HRP)-like properties, dissolving the long-held belief that inorganic materials are biologically inert, and opening the field of nanozyme research (Fan et al., 2020a). Any nanozyme, a nanomaterial with enzymatic activity, is classified into two categories–a nanomaterial modified to associate with a natural enzyme or a group of enzymes or nanomaterials that have enzymatic properties (Dutta et al., 2013; Wan et al., 2014). Compared to natural enzymes, nanozymes have advantages, such as enhanced stability, regulable activity, high recycling efficiency, etc., which are helpful in the detection of the safety of use, disposal, disease surveillance, and biomedicine (Asati et al., 2009). The past decade has witnessed the development of several nanozymes, such as CeO_2_ (Karakoti et al., 1989), Prussian blue (PB) (Zhang et al., 2016), Pt (Zhang et al., 2010), Pd (Ge et al., 2016), etc., leading to a boom in nanomedicine.

**FIGURE 2:**
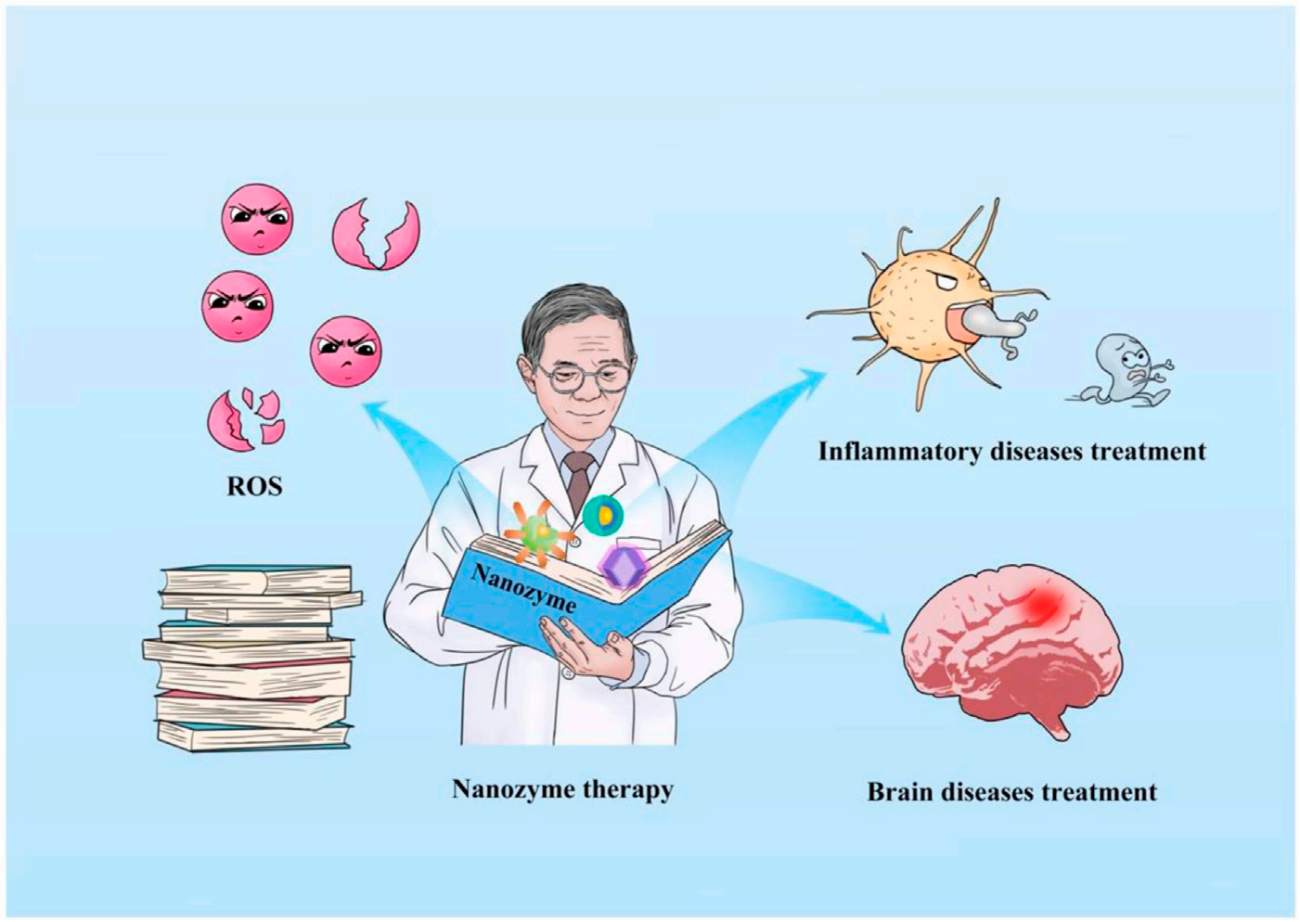
Schematic illustrating the review of nanozyme-based nanotherapy for eliminating ROS and regulating redox homeostasis, ultimately treating inflammation and brain diseases.

Despite the extensive reviews on the use of nanozymes in the treatment of disease, only a handful of them have emphasized the maintenance of healthy redox levels. Considering the significant progress in the last 5 years, especially in ROS-eliminating nanozymes (Figure 2), in this review, we discussed the development and applications of nanozymes that regulate the levels of ROS in the treatment of ROS-induced diseases. The focus is laid on inflammation and brain diseases caused by excess ROS and the nanozyme-therapy strategies developed recently.

## Nanozyme-Based Treatment of Inflammation

### Nanozyme-Based Treatment of Inflammatory Bowel Disease

Inflammation is the response by the immune system of vertebrates to foreign harmful factors, including pathogens, which are associated with several diseases including rheumatoid arthritis, obesity, and cancer (Zhang et al., 2021b). Although induced by exogenous stimuli, the dysregulation of ROS plays versatile roles in the pathogenesis. Therefore, it is believed that the antioxidant activity of nanozymes will be helpful in the treatment of inflammation. For instance, as reported by (Li et al., 2016) platinum-doped Prussian blue nanoparticles (PPBs) with ROS-scavenging properties relieved inflammation induced by tumor photothermal therapy (PTT) (Li et al., 2021a).

Inflammatory bowel disease (IBD), as a refractory chronic disease, represents the kind of autoimmune disorders in which the immune system attacks the digestive system (Maloy and Powrie, 2011; Hoivik et al., 2012). Although the fatality rate of IBD is low, the quality of life of patients deteriorates. Traditional remedies for IBD like antibiotics and antibodies cause complications like antibiotic resistance, creating a need for research and the development of novel drugs (Vong et al., 2012; Takedatsu et al., 2015). Because most drugs for IBD are taken orally, their stability is of primary concern. Therefore, Zhu et al. (2017) developed Selenium (Se) nanoparticles modified by Ulva lactuca polysaccharide (ULP) to improve the stability of Se. Se is a widely accepted nutritional antioxidant that has implications in human disorders, especially IBD. Besides, Song et al. designed Se nanoparticles with Kluyveromyces lactis GG799, which could transform sodium selenite to Se nanoparticles (Song et al., 2021). The study results suggested that both ULP and Kluyveromyces lactis GG799 decorated Se nanoparticles could alleviate oxidative stress and the inflammatory response, thus offering promising therapeutic strategies for acute colitis.

Moreover, nanozymes present a promising strategy for the treatment of IBD due to their high catalytic activity *in vivo* (Vong et al., 2015; Zhu et al., 2017). PPBs can be used as theranostics due to their high magnetic and enzymatic properties (Zhang et al., 2017). Zhao et al. (2018) reported that polyvinylpyrrolidone (PVP)-modified PPBs with multi-enzyme properties and biosafety improved DSS-induced IBD (Kong et al., 2015). In 2019, Zhao et al. (2018) optimized the therapeutic system by introducing manganese (Mn). Due to the low redox potential of Mn (II), the activity of the nanoplatform was significantly improved. Additionally, the Mn PPBs are adsorbed onto the inflamed mucosa electrostatically. The nanozyme activated the toll-like receptor (TLR) signaling pathway, exerting a synergistic effect of ROS-depletion and TLR-activation to improve DSS-induced colitis. When it comes to targeted therapy to the intestines, negatively charged montmorillonite (MMT), which is a clinically approved drug that preferentially accumulates onto the positively charged inflamed mucosa, is used. Zhao et al. (2019) combined MMT with CeO_2_ using the *in situ* growth strategy to construct CeO_2_@MMT. After oral administration, the negatively charged CeO_2_@MMT specifically targeted the positively-charged inflamed colon and scavenged ROS by binding to it electrostatically. Therefore, the pro-inflammatory macrophages (M1) and cytokines decreased while the anti-inflammatory macrophages (M2) and IL-10 increased, thus, repairing the injured intestinal epithelium and increasing the length of the colon of mice.

**FIGURE 3:**
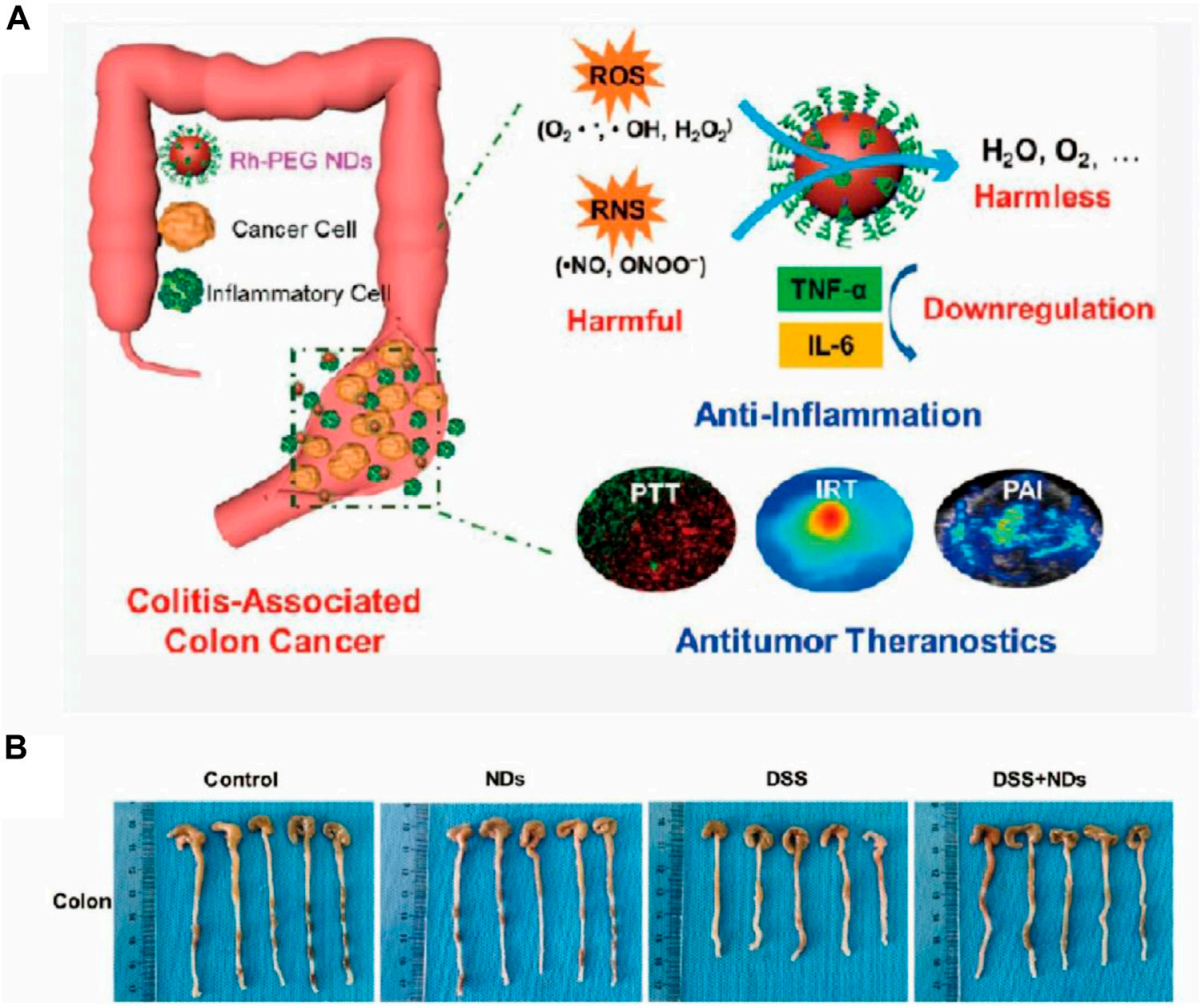
**(A)** Schematic of PEG-coated ultrasmall rhodium nanodots with RONS eradicating and photothermal activities for IBD and tumor theranostics *in vivo*. **(B)** Photographs of colons of various groups showing the anti-inflammatory treatment of colitis. Reproduced from Coussens and Wern (2002) with permission from the American Chemical Society.

Several reports suggested that nanozymes produced superfluous ROS, mainly attributed to their peroxidase (POD)-like activity, thus acting as a therapeutic for inflammation caused by bacterial infection (Wang et al., 2017; Sun et al., 2020; Zhao et al., 2020; Huang et al., 2021). In the report by Fan et al. (2020b) related to the treatment of both bacteria-infected wounds and IBD (Shi et al., 2018), Fe- and N-doped hollow carbon spheres were constructed using a one-pot strategy. The proposed nanozyme was successfully used against both infectious and noninfectious inflammation due to its POD-like and ROS-scavenging properties, respectively, shedding light on the importance and possibility of synthesizing nanozymes with multi-enzyme properties for the treatment of inflammation. In addition to the individual application of multi-enzyme-mimetic properties of nanozymes in various therapies, the use of a cascade of catalytic reactions in a specific integrated system has also been reported. Liu et al. developed an integrated nanozyme (designated as Pt@PCN222-Mn) to remove ROS for the remission of IBD, where Mn (III) porphyrin showed SOD-like property and platinum (Pt) nanoparticles showed CAT-like property (Fan et al., 2020b). Through the synergistically enhanced ROS-eliminating effect *in vivo*, the nanozyme showed great therapeutic potential in ROS-related IBD and broadened the possibility for the design of more cascade nanozymes. IBD promotes other metabolic disorders throughout the gastrointestinal system, leading to more lethal diseases like colitis-associated colorectal cancer (CAC) (Liu et al., 2020a). With this in mind, Miao et al. constructed versatile ultrasmall rhodium nanodots coated with polyethylene glycol (PEG), to treat inflammation and cancer using the ROS-eliminating effect and the photothermal performance of the nanozyme (Figure 3A) (Coussens and Werb, 2002). The *in vivo* results demonstrated desirable therapeutic effect of colitis, managing the bowel disease with high efficiency (Figure 3B).

### Nanozyme-Based Treatment of Acute Kidney Injury

Acute kidney injury (AKI) is another disease related to inflammation and ROS, clinical symptoms of which include renal insufficiency, increased metabolite accumulation, and perturbation of acid-base homeostasis (Miao et al., 2020). Amifostine (AMF), which promotes free radical scavenging, has been used to treat AKI. Although side effects of AMF limit its wider application, the radical-based strategy opened a novel therapeutic window to treat AKI (Dennis and Witting, 2017). Several studies have shown that AKI patients exhibited excessive oxidative stress, ultimately leading to renal dysfunction (Bukowski, 1996). A majority of antioxidants against AKI cannot pass through the glomerulus. Therefore, a group of researchers designed a series of ultrasmall nanozymes. As an example, Liu et al. (2020b) developed RuO_2_ nanozymes with an average size of about 2 nm, which passed through the glomerulus and was excreted. AKI in mice was significantly alleviated due to the antioxidant activity of RuO_2_ (Loynachan et al., 2019). Similar ceria-based nanoparticles were developed by Zhang et al., 2020 Desirable results were obtained for rhabdomyolysis-induced AKI due to the ultrasmall size and multi-enzymatic properties of the nanozyme (Liu et al., 2020b). Apart from AKI, ultrasmall nanozymes may be used to treat other ROS-associated diseases due to their rapid renal clearance and biocompatibility. Liu et al. (2020c) reported an ultrasmall-sized Cu_5.4_O nanozyme exhibiting broad-spectrum ROS-eliminating effect to treat AKI, acute liver injury (ALI), and wound healing (Zhang et al., 2020). The transcriptomics analysis revealed that the nanozyme upregulated the oxidative stress-related genes, subsequently activated the MAPK signaling pathway, and alleviated AKI. It may be used to treat other oxidative stress-induced disorders.

### Nanozyme-Based Treatment of Other Inflammatory Diseases

**FIGURE 4:**
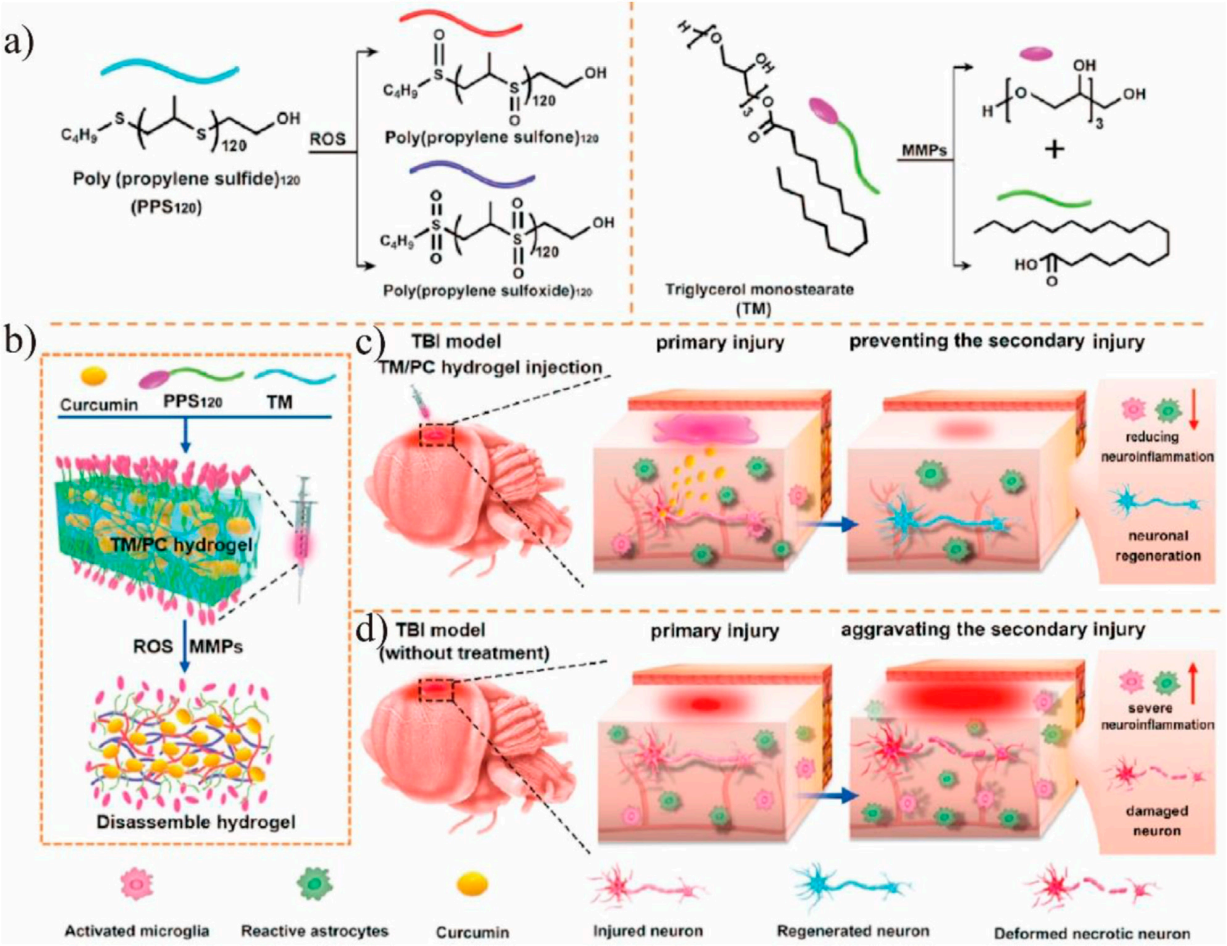
**(A)** PPS_120_ switched from a hydrophobic polymer to the hydrophilic poly (propylene sulfone)_120_ and poly (propylene sulfoxide)_120_ in the ROS environment and TM could be cleaved by MMPs. **(B)** Schematic illustration of TM/PC hydrogel preparation procedures and their degradation process under MMP and ROS conditions. **(C)** *In situ* injection of TM/PC hydrogels within the postsurgery TBI: TM is degraded, and PPS120 scavenges ROS to release Cur, reducing neuroinflammation and neuronal death. **(D)** Without hydrogel treatment, neuronal death and severe neuroinflammation were observed, and the secondary injury was aggravated. Reproduced from Qian et al. (2021) with permission from the Elsevier.

Sepsis is a kind of severe systemic disease caused by the entry of pathogenic bacteria and their toxins into the bloodstream (Liu et al., 2020c). Excessive ROS plays a vital role in the occurrence of sepsis. Chen et al. (2021) reported a new selenium-hyaluronic acid (HA) nanozyme for the treatment of sepsis injury, where the introduction of HA not only provided the nanozyme with an inflammatory macrophage-targeting property but also enhanced the ROS-eliminating capacity of the nanocomposites (Yang et al., 2019a). Vascular restenosis, a major problem in endovascular interventional therapy, is also associated with ROS. Feng et al. (2020) constructed the Prussian blue nanozyme, in combination with a novel therapy called vascular balloon injury (VBI), which facilitated the entry of the nanozyme across the vascular intima and uptake by macrophages to alleviate vascular restenosis (Chen et al., 2021). Cardiac ischemia-reperfusion (CIR) injury is one of the most complicated diseases and its underlying mechanisms are not clear. With its characteristic feature of excess ROS in the mitochondria in mind, Zhang et al. developed a hybrid nanozyme consisting of a protein scaffold and a metal nanoparticle core that harbored both mitochondria-targeting and ROS-removal properties (Li et al., 2021a). The *de novo* design strategy in artificial enzyme synthesis shed light on the development of nanozyme and ameliorated oxidative injury in CIR. Tobacco use has threatened health globally. In addition to nicotine and tar, ROS like radicals and hydrogen peroxide are other lethal factors that cannot be efficiently removed by cigarette filters. Lin et al. (2020) reported a copper tannic acid (CuTA)-associated nanozyme to improve the cigarette filters by its antioxidant effect (Figure 4A) (Feng et al., 2020). Certain *in vivo* studies demonstrated that the nanozyme could scavenge oxidative stress in the cigarette efficiently, alleviating ROS-associated lung inflammation and acute lung injury (Figures 4B–E). In organisms, several enzymes are involved in specific life processes. One of these processes is the antioxidant system. To mimic the antioxidant system in cells, Yao et al. (2018) synthesized Mn_3_O_4_ that showed multiple enzymatic properties and used it for the treatment of ROS-induced ear inflammation (Lin et al., 2020). Bao et al. (2018) used polydopamine nanoparticles as ROS scavengers for the first time in the treatment of oxidative stress-related periodontal disease. In murine periodontitis models, the polydopamine nanoparticles decreased ROS and inflammation (Yao et al., 2018).

## Treatment for Brain Diseases Based on Nanozymes

### Treatment for Traumatic Brain Injury Based on Nanozymes

Brain diseases, including brain injury, Alzheimer’s disease (AD), Parkinson’s disease (PD), etc., implicate the involvement of ROS and redox imbalance. For example, traumatic brain injury (TBI), which may lead to permanent impairment of the nervous system, consists primarily of brain damage caused by an accident and secondarily brain injury by oxidative damage to proteins, lipids, and nucleic acids due to ROS (Simon et al., 2017; Bao et al., 2018). Nagasaki’s team found that by orally administration of redox polymers with antioxidant nitroxide radicals, the cognition ability in senescence-accelerated prone (SAMP8) mice was significantly improved due to the elimination of ROS (Chonpathompikunlert et al., 2015). The blood-brain barrier (BBB) is a unidirectional, selective, and concentration-dependent barrier protecting the brain parenchyma. Poor delivery to the brain is the main obstacle for the nanodrugs in the treatment of brain diseases. Various nanozymes have been developed for the brain diseases treatment (Table 1). Large nanoparticles are susceptible to capture by the reticuloendothelial system, resulting in the availability of only a few drugs. Therefore, ultrasmall and targeted nanozymes and non-invasive therapies are developed. For example, He et al. (2020a) suggested ultrasmall nanoparticles that aggregated under ROS-rich conditions. Studies found that the nanozyme had a nearly 9-times-higher uptake compared to other nanozymes and preferred to aggregate under ROS-rich conditions in the mitochondria, thus, having great potential for ameliorating ROS damage in TBI. To efficiently scavenge reactive oxygen and nitrogen species (RONS) in TBI, Mu et al. (2019a) constructed a carbogenic small-sized nanozyme. Due to its selective elimination of RONS and ultrahigh enzymatic activity, the nanozyme was successfully used in both *in vitro* experiments and TBI mice. Nanozymes with a preference for the physiological environment are crucial for disease treatment, especially for brain diseases. However, most nanozymes do not exhibit optimal activity under physiological conditions. To enhance the catalytic selectivity of nanozymes, Mu et al. (2019b) designed trimetallic nanozymes with multi-antioxidative properties and no environment preference. The nanozymes eliminated the excess free radicals in the H_2_O_2_-treated neural cells and the injured brain tissue, thus, decreasing the lethality of the brain injury. Given that most nanoparticles have difficulties in passing the BBB, non-invasive treatment is a desirable alternative therapy for brain diseases. Qian et al. (2021) developed an *in situ* implantable, ROS-scavenging, and post-trauma responsive hydrogels for the treatment of TBI (Figure 4A). After injecting into the surgical cavity after TBI, the poly (propylene sulfide) 120 component of the hydrogels switched from a hydrophobic polymer to a hydrophilic one in the ROS-rich environment, and the triglycerol monostearate (TM) was cleaved by matrix metalloproteinases (MMP), releasing the embedded curcumin to reduce the ROS levels (Figure 4B). Further research suggested that the hydrogels decreased inflammation and promoted neuronal regeneration while maintaining the integrity of the BBB (Figures 4C,D). The traditional compression bandages have antioxidative properties and reduce neuroinflammation and mitigate the symptoms of TBI to some degree. As a result of the unfavorable electron-donating ability, the bandage possesses low recycling efficiency (Sood et al., 2014). Yan et al. (2019) reported a kind of bandage based on single-atom nanozyme, namely Pt/CeO_2_, which provided a non-invasive and persistent treatment for TBI. *In vivo* studies showed that the catalytic activity of the nanozyme lasted for a month, considerably improving wound healing after TBI.

**TABLE 1:** A brief summarize of the applications of nanozyme in brain diseases.

Types of brain diseases	Ligands	Main metal elements	Surface modification	References
TBI	GSH, Lys	C, S, O, N	—	He et al. (2020a)
	Ascorbic acid, Lys	C	—	Mu et al. (2019a)
	PVP	Ru	—	Wu et al. (2021)
AD	Erythrocyte membrane	Cu	Targeting peptide KLVFF	Ma et al. (2020)
	Retinoic acid	Ce	MOF	Yu et al. (2020)
PD	PVP	Cu, Pt	—	Liu et al. (2021)
	GSH	Ce	Er^3+^, Yb^3+^	Li et al. (2021a)
Ischemic stroke	PVP	Ce	ZIF-8	He et al. (2020b)
	PVP	Fe	Neutrophil-like cell Membrane	Feng et al. (2021)

### Treatment for Alzheimer’s Disease Based on Nanozymes

**FIGURE 5:**
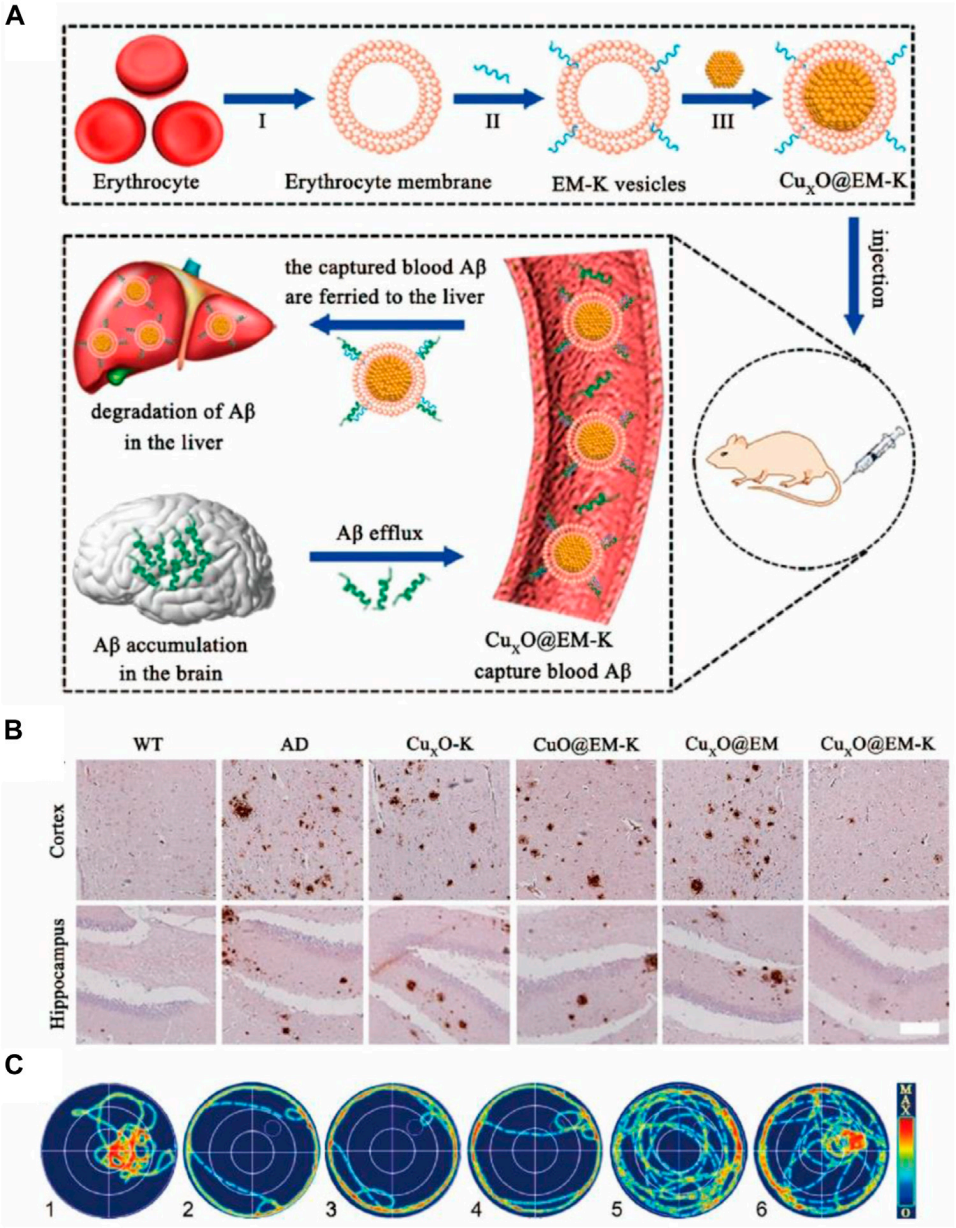
**(A)** Preparation of CuxO nanozyme wrapped with erythrocyte membrane (CuxO@EM-K). The resulting CuxO@EM-K captures A*β* in the blood followed by elimination of A*β* bound to CuxO@EM-K by the liver, facilitating efflux of A*β* from the brain into blood through the sink effect and leading to the reduction A*β* burden in brain. **(B)** Representative images of A*β* staining in both the cortex and the hippocampus. Scale bar: 100 μm. **(C)** Representative swimming paths of mice in the probe test. Reproduced from Ma et al. (2020) with permission from the American Chemical Society.

Alzheimer’s disease (AD), the most common neurodegenerative disease in the world, is characterized by loss of cognition and memory. Millions suffer from the disease and the number is predicted to reach one hundred million by 2050 (Sweeney et al., 2018). Mounting evidence suggested that accumulation of amyloid-*β* (A*β*), ROS, and neuronal loss are the major causes underlying the pathological manifestations in AD. Nanozymes with simulated enzymatic properties were used to counter the above three causal factors and they ameliorated the symptoms of AD (Wood, 2015; Ordóñez-Gutiérrez et al., 2015). As an example, Guan et al. (2016) and Gao et al. (2021) from Qu’s team developed two kinds of nanozymes based on polyoxometalates, namely CeONP@POMD and AuNPs@POMD, respectively, and both these nanozymes showed proteolytic and SOD-like properties. CeONP@POMD inhibited the activation of microglial cells and promoted the proliferation of PC12 cells, which were results of the A*β*-degrading and ROS-depleting effects of the nanozyme. Likewise, AuNPs@POMD’s protease-like activity was used to inhibit the aggregation of A*β*, while the SOD-like activity was helpful in scavenging ROS, the production of which was mediated by A*β*. Also, both the nanozymes passed through the blood-brain barrier (BBB) and exhibited low toxicity. Instead of decreasing the A*β* using BBB-permeable nanozymes, it is nanozyme wrapped with erythrocyte membrane to overcome the interference of protein corona formation and immune responses in nanomedicine (Ma et al., 2020). An A*β*-targeting peptide KLVFF was attached to the erythrocyte membrane so that it could selectively capture A*β* in the blood (Figure 5A). *In vivo* experiments suggested that the nanocomposites could promote liver degradation by A*β* and mitigate membrane oxidative damage induced by A*β* (Figure 5B), thus alleviating the memory deficits in AD mice by decreasing peripheral A*β* burden (Figure 5C). As discussed above, A*β*-induced ROS damage may compromise neurogenesis in AD patients, and neuronal loss is considered the ultimate cause of pathological damage in AD. With this in mind, Yu et al. (2020) reported a ROS-responsive metal-organic framework (MOF) loaded with small interfering RNA (siSOX9), retinoic acid (RA), and CeO_2_. Benefiting from siSOX9 and RA, the neural stem cells (NSC) differentiated into neurons with high efficiency (Yu et al., 2020). CeO_2_ in the MOF helped avoid oxidative damage and guaranteed an improved desirable to clear the peripheral A*β* to treat AD. Ma et al. (2020) designed the Cu_x_O survival rate of the newly differentiated neurons. Experiments using an AD mouse model suggested that the rational-designed nanoparticles significantly promote dneurogenesis and mitigated the cognitive impairment of triple transgenic AD mice.

### Treatment for Parkinson’s Disease Based on Nanozymes

**FIGURE 6:**
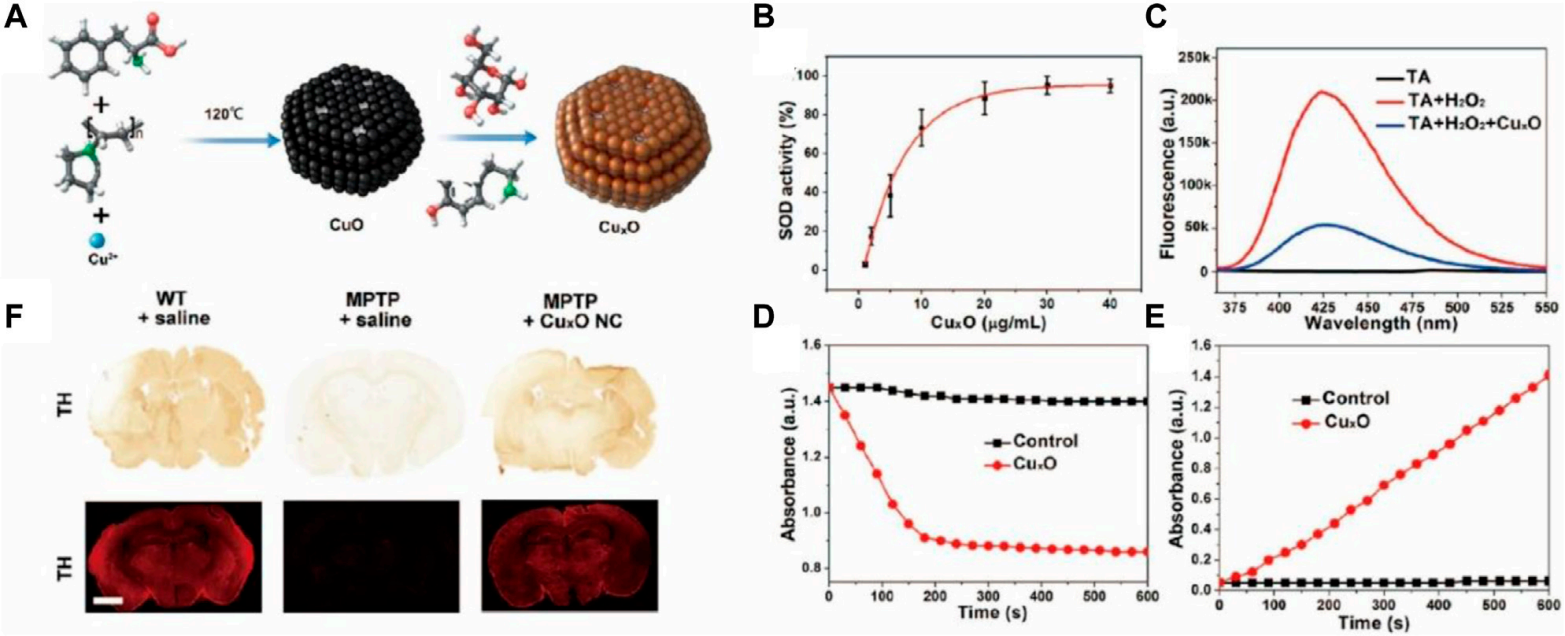
**(A)** Schematic showing the preparation of CuxO. CuxO exhibits multienzyme-likeactivities. **(B)** SOD-like activity, **(C)** CAT-like activity, **(D)** GPx-like activity, **(E)** POD-like activity. **(F)** Immunohistochemistry (IHC) images and immunohistofluorescence (IHF) images of tyrosine hydroxylase (TH) in the brains of control, MPTP-induced PD mice, and CuxO NCs-treated mice. Scale bar: 1 mm. Reproduced from Singh et al. (2017) with permission from the American Chemical Society.

Parkinson’s disease (PD), also known as “palsy tremor,” is the second most common neurodegenerative disease in the world, affecting nearly 10 million people worldwide (Jeong et al., 2019). Among people over 60 years of age, the average incidence of PD is about 1% (Dani et al., 2020; Song et al., 2021). Due to the diverse and complex causes of PD, the treatment of PD is laden with difficulties, and the current therapeutic drugs have serious side effects, resulting in complications. There is evidence that ROS causes neural dysfunction due to oxidation caused by mitochondrial dysfunction, providing a novel idea for the treatment of neurodegenerative diseases such as PD (Kwon et al., 2013). However, natural antioxidants are sensitive to physiological conditions. Therefore, the development of nanozymes for PD therapy has attracted increasing attention by researchers, especially to develop nanozymes with a higher and broader spectrum of antioxidant properties. CeO_2_ is a widely used antioxidant nanozyme. However, CeO_2_ has limitations in PD treatment due to its ROS-catalytic properties and unfavorable BBB permeability. Therefore, Li et al. doped Yb^3+^ and Er^3+^ ions onto CeO_2_ nanoparticles, increasing oxygen vacancy and, thus, leading to higher catalytic properties (Liu et al., 2014). Singh et al. developed Mn_3_O_4_ nanozymes with multi-enzymatic properties that were remarkably higher than the other available nanozymes including Fe_3_O_4_, CeO_2_, and V_2_O_5_ (Li et al., 2021b). The developed Mn_3_O_4_ nanoparticles had a desirable protective role in MPP^+^-induced nerve cell injury through its redox modulatory effect, thus, showing a great promise in the prevention of ROS-mediated PD in a disease model. Hao et al. (2019) prepared Cu_x_O nanozymes associated with phenylalanine (Phe) as a structure-directing agent (Figure 6A) (Singh et al., 2017). Due to their small size (about 65 nm) and enzyme-mimicking properties (including superoxide dismutase, catalase, and glutathione peroxidase, etc.) (Figures 6B–E), the nanozyme exhibited neuroprotective effects in mice with PD (Figure 6F). Wu et al. (2021) developed ultrasmall ruthenium oxide nanozymes that could simultaneously imitate multi-enzymatic activities, protect proteins and lipids from oxidative damage by UV or H_2_O_2_, and exert substantial remission in inflammation and PD symptoms (Hao et al., 2019). To the best of our knowledge, abnormal aggregation of *α*-synuclein (*α*-syn) is one of the main causes of PD. Braak proposed that *α*-syn may spread in the PD brain, which subverted the fundamental theory of PD. Moreover, despite direct nerve damage caused by ROS, the latter can also contribute to the spread of pathogenic *α*-syn by inducing oxidative stress. With this in mind, Liu et al. (2021) proposed PtCu nanoalloys (NAs) with ROS-eliminating properties to counter *α*-syn transmission (Wu et al., 2021). This provided evidence for the inhibitory effect of the nanozymes on the spread of *α*-syn across neurons.

### Treatment for Other Brain Diseases Based on Nanozymes

Ischemic stroke is the most common type of stroke leading to disability in a significant proportion of the population in the 21st century. Although clinical interventions available may realize recanalization of blood vessels after blockage by a thrombus, overproduction of ROS during the process of reperfusion may cause secondary damage to the cerebrovascular system and neural tissues. Moreover, reperfusion causes significant damage due to an inflammatory response generated by ROS (Andrabi et al., 2019). Nanotechnology-mediated therapy has exhibited some success in the treatment of ischemia-reperfusion injury after ischemic stroke. For instance, He et al. (2020b) developed a zeolitic imidazolate framework-8 (ZIF-8)-capped CeO_2_ nanozyme using *in situ* synthetic strategy, which inactivated astrocytes and suppressed the secretion of inflammatory cytokines due to their catalytic and antioxidative properties, proving to be a therapy for ischemic stroke. To improve the amount of drug delivered to the brain lesion tissue, Feng et al. (2021) developed PPBs coated with a neutrophil-like cell membrane to realize the active-targeting treatment for ischemic stroke. The nanozymes accumulated in the damaged brain and the inflamed brain endothelial cells benefited from the innate targeting properties of neutrophils. Additionally, long-term therapeutic efficacy of the composites was examined in detail and the underlying mechanisms, such as microglial polarization and neural stem cell proliferation, were uncovered.

## Challenges of Nanozymes in Biomedical Applications

Although much efforts have been made to enhance the biocompatibility and reduce the toxicity of nanoparticles, it is still a thorny problem for researchers. As an instance, there are no effective strategies to reduce the toxicity of cobalt nanoparticles (CoNPs) in clinical applications. To make a comprehensive understanding of CoNPs toxicity to benefit the design of safe detoxification drugs, Wang et al (2011), studied the effect of nano-selenium (BNS) in inhibiting the toxicity of CoNPs and found that BNS could reduce the ROS and inflammatory respond elicited by CoNPs via the KNA signaling pathway, thus antagonizing CoNPs-toxicity efficiently.

The pharmacokinetics and biodistribution of therapeutic nanozymes has vital implications for their applications *in vivo*, and have been investigated with great efforts. For example, TiO2 nanoparticles, Pt nanoparticles, selenium nanoparticles and CeO2 have been systematically studied for their biological behaviors after administration (De Oliveira et al., 2017; Pham et al., 2018). Vong et al. (2012) developed a nitroxide radical-containing nanoparticles (RNP^o^) with a diameter of 40 nm, which could avoid being absorbed into the bloodstream, thus having a desirable distribution in the colonic mucosa and effectively preventing its accumulation in other organs. Therefore, no obvious toxicities could be found *in vivo* even after multiple oral administration of RNP^o^. When combined with irinotecan, an kind of chemotherapy agent, obvious therapeutic effect of enteritis and colon cancer could be observed (Vong et al., 2015). Besides, to investigate the time-dependent accumulation of TiO_2_ in various organs, Elgrabli et al. (2015) constructed a pharmacokinetic model, and found that TiO_2_ with a diameter below 25 nm could be eliminated from the body efficiently with a half-life of 12.7 days.

It’s important to give an in-depth interpretation in the manners by which these nanoparticles interact with ROS and the biodegradability of nanoparticles in targeted sites. Typically, nanomaterials will be patrolled by immune system and regarded as extraneous invaders. Subsequently, oxidant-generating enzymes will be expressed to generate excess ROS for nanoparticle disintegration. Organic nanoparticles usually have desirable degradability in respond to ROS. As an instance, Kwon et al. (2013) designed a ROS-responsive polymeric prodrug poly (vanillin oxalate) (PVO), which could degrade into antioxidant vanillin under oxidative damage environment.However, inorganic nanomaterials have higher stability, thus needing more ingenious designs to improve their ROS-response and degradation ability. The application of redox-active moieties in the design of inorganic nanomaterials has been widely accepted as a method to enhance oxidative biodegradability (Liu et al., 2017). Nevertheless, the interactions between ROS and the biodegradability of those nanoparticles is still unclear (Walia et al., 2017).

## Summary and Perspectives

Unique ROS-regulatory properties of the nanozymes have helped researchers fulfill the ever-stringent requirements in medicine. To date, varieties of nanozymes have been developed for ROS-related diseases, such as IBD, AKI, TBI, PD, AD, et al. Even then, research on nanozymes is still in the initial stages, leaving a substantial amount of scientific or technological issues to be addressed.

1) Cytotoxicity is often initiated due to the inorganic nature and metal ions involved in nanozymes. Most of the metal ions used to develop nanozymes are not essential to organisms. Recent research suggested that some inorganic components in nanozymes also release ROS, which might compromise the therapeutic effect of the nanozymes (Yang et al., 2019b). Moreover, a majority of nanozymes cannot be degraded in the biological milieu, which may result in sustained ROS production and acute cytotoxicity. Therefore, to minimize the damage to normal tissues, precision medicine, which represents a new era of disease therapy, is critical for the optimization of therapeutic outcomes. Quite a few nanozymes are being developed with targeted abilities to meet the requirements of precision medicine, such as specific ligand modifications or membrane encapsulations (Qin et al., 2020).
2) Having multi-enzyme-mimetic properties is one of the main features of nanozymes, often being considered to be associated with their prominent therapeutic effects and versatility in the medical field. However, this characteristic can result in insufficient catalytic activity in some specific reactions. Compared to natural enzymes and organic catalysts, the catalytic efficiency of nanozymes is relatively low. Therefore, researchers are trying to improve the activity of nanozymes by adjusting the size and composition, modifying the surfaces, doping ions, and so on. Moreover, inspired by the recent advances in catalytic chemistry, single-atom nanozymes have been developed that improve catalytic efficiencies and regulate ROS *in vivo*. The improvement in catalytic properties might reduce the drug dosages given to the patients, decreasing the cytotoxicity, which is another hard nut to crack regarding the use of nanozymes, as discussed above.
3) Despite increased research on nanozymes over the years, clinical translation has encountered bottlenecks, and only a few nanozymes have been approved and commercialized. Quite a few reasons contributed to these bottlenecks. Firstly, given the superior therapeutic outcomes in established animal models, the biological mechanisms used by these nanozymes have not been successfully elucidated. Also, the versatility of ROS in organisms makes it difficult to administer appropriate doses of nanozymes that will ensure therapeutic effects and limit pathological changes. Lastly, it is difficult to select the optimized nanozymes for the subsequent clinical trials from the increasing amounts of newly developed ROS-based nanozymes. In response to the above questions, more in-depth mechanistic research, such as the elucidation of the underlying intracellular signaling pathways and the interaction of the antioxidant nanozymes with the *in vivo* environment, should be performed. In addition, more rigorous efficacy and safety evaluation should be accompanied in studies reporting the effects of nanozymes, which might benefit the screening process of the most effective nanozymes for follow-up clinical research.

As further research addressing the above-mentioned issues related to nanozymes involved in ROS-scavenging is performed, nanozymes are expected to be promising candidates contributing to human health and well-being.
